# Distinct disease features in chimpanzees infected with a precore HBV mutant associated with acute liver failure in humans

**DOI:** 10.1371/journal.ppat.1008793

**Published:** 2020-08-31

**Authors:** Zhaochun Chen, Ronald E. Engle, Chen-Hsiang Shen, Huaying Zhao, Peter W. Schuck, Emily J. Danoff, Hanh Nguyen, Norihisa Nishimura, Kevin W. Bock, Ian N. Moore, Peter D. Kwong, Robert H. Purcell, Sugantha Govindarajan, Patrizia Farci

**Affiliations:** 1 Hepatic Pathogenesis Section, Laboratory of Infectious Diseases, National Institute of Allergy and Infectious Diseases, National Institutes of Health, Bethesda, Maryland, United States of America; 2 Vaccine Research Center, National Institute of Allergy and Infectious Diseases, National Institutes of Health, Bethesda, Maryland, United States of America; 3 Laboratory of Cellular Imaging and Macromolecular Biophysics, National Institute of Biomedical Imaging and Bioengineering, National Institutes of Health, Bethesda, Maryland, United States of America; 4 Infectious Disease Pathogenesis Section, National Institute of Allergy and Infectious Diseases, National Institutes of Health, Bethesda, Maryland, United States of America; 5 Department of Pathology, University of Southern California, Los Angeles, California, United States of America; Albany Medical College, UNITED STATES

## Abstract

Transmission to chimpanzees of a precore hepatitis B virus (HBV) mutant implicated in acute liver failure (ALF) in humans did not cause ALF nor the classic form of acute hepatitis B (AHB) seen upon infection with the wild-type HBV strain, but rather a severe AHB with distinct disease features. Here, we investigated the viral and host immunity factors responsible for the unusual severity of AHB associated with the precore HBV mutant in chimpanzees. Archived serial serum and liver specimens from two chimpanzees inoculated with a precore HBV mutant implicated in ALF and two chimpanzees inoculated with wild-type HBV were studied. We used phage-display library and next-generation sequencing (NGS) technologies to characterize the liver antibody response. The results obtained in severe AHB were compared with those in classic AHB and HBV-associated ALF in humans. Severe AHB was characterized by: (i) the highest alanine aminotransferase (ALT) peaks ever seen in HBV transmission studies with a significantly shorter incubation period, compared to classic AHB; (ii) earlier HBsAg clearance and anti-HBs seroconversion with transient or undetectable hepatitis B e antigen (HBeAg); (iii) limited inflammatory reaction relative to hepatocellular damage at the ALT peak with B-cell infiltration, albeit less extensive than in ALF; (iv) detection of intrahepatic germline antibodies against hepatitis B core antigen (HBcAg) by phage-display libraries in the earliest disease phase, as seen in ALF; (v) lack of intrahepatic IgM anti-HBcAg Fab, as seen in classic AHB, but at variance with ALF; and (vi) higher proportion of antibodies in germline configuration detected by NGS in the intrahepatic antibody repertoire compared to classic AHB, but lower than in ALF. This study identifies distinct outcome-specific features associated with severe AHB caused by a precore HBV mutant in chimpanzees, which bear closer resemblance to HBV ALF than to classic AHB. Our data suggest that precore HBV mutants carry an inherently higher pathogenicity that, in addition to specific host factors, may play a critical role in determining the severity of acute HBV disease.

## Author summary

While the pathogenesis of classic acute hepatitis B (AHB) is believed to be mediated by hepatitis B virus (HBV)-specific T-cell responses, the pathogenesis of HBV-associated acute liver failure (ALF), one of the most rapid and lethal liver diseases, remains largely unknown. Our previous work demonstrated that ALF is associated with an uncommon B-cell response with the intrahepatic production of antibodies in germline configuration directed against the hepatitis B core antigen (HBcAg). Previous reports also documented an association of ALF with HBV variants containing precore or core promoter mutations. Here, we studied chimpanzees experimentally infected with a precore HBV mutant implicated in human ALF with the aim of getting new insights into ALF pathogenesis. Although these chimpanzees did not develop ALF, they had an unusually severe AHB with distinct disease features, including the highest alanine aminotransferase (ALT) peaks ever seen in HBV transmission studies, a shorter incubation period with an earlier ALT peak, an earlier HBsAg clearance and antibody seroconversion compared to classic AHB, and intrahepatic B-cell infiltration. Remarkably, the ALT peak correlated with extensive hepatocellular damage, but very limited inflammatory reaction. As previously documented in ALF, anti-HBcAg antibodies in germline configuration were present in the liver in the earliest disease phase. Thus, although chimpanzees did not develop ALF, infection with a precore HBV mutant caused a severe AHB with several analogies with ALF, suggesting that precore HBV mutants carry an inherently higher pathogenicity.

## Introduction

The pathogenesis of HBV-associated acute liver failure (ALF), previously termed fulminant hepatitis B, is still largely unknown[1, 2]. Early studies had documented an unusually brisk antibody response to HBV antigens and a more rapid viral clearance compared to classic acute hepatitis B,[3–5] but the role of these findings in the pathogenesis of HBV ALF has long remained undefined. Moreover, ALF has been associated with infection by HBV variants containing precore or core promoter mutations [6–8], but these mutations are also frequently detected in asymptomatic hepatitis B surface antigen (HBsAg) carriers and in patients with chronic hepatitis B [9]. Recently, we provided evidence that HBV ALF is associated with a T-cell independent, intrahepatic B-cell response with extensive production of antibodies in germline configuration that recognize the hepatitis B core antigen (HBcAg) with subnanomolar affinity [10]. Next-generation sequencing (NGS) of HBV strains isolated from the liver of patients with HBV ALF showed a highly mutated HBcAg with the precore stop codon mutation present in 100% of the viral population. Thus, our previous study delineates ALF as an anomalous HBV core-driven B-cell disease [10], which results from the encounter between a highly mutated HBcAg and an unusual B-cell response.

The chimpanzee model has played a major role in the study of acute hepatitis B [11, 12], but attempts to transmit hepatitis B virus from patients with fulminant hepatitis B never resulted in ALF, which is one of the most dramatic clinical syndromes characterized by a sudden loss of hepatocytes associated with coaugulopathy and encephalopathy in individuals without preexisting liver disease [1]. However, experimental transmission of a precore HBV mutant to chimpanzees, implicated in fulminant hepatitis B in humans, resulted in an unusually severe acute hepatitis compared to the classic acute hepatitis B (AHB) following inoculation with the wild-type of HBV [11, 13, 14], but not in the development of ALF [15]. Access to archived serum and liver specimens from these chimpanzees previously infected with a precore HBV mutant that caused fatal ALF in humans [15], provided us with the opportunity to characterize the clinical, serologic and virologic profile, and the host immune response of severe AHB and to compare the results with those of classic AHB and HBV-associated ALF in humans. By using phage-display technology and NGS we characterized the intrahepatic antibodies response against HBcAg and the global liver antibody repertoires. The results of this study identify distinct-outcome specific features that allowed us to differentiate severe from classic AHB and shed new insights into the pathogenesis of acute HBV infection caused by a precore HBV mutant.

## Results

### Clinical, serologic and virologic characteristics in severe versus classic AHB in chimpanzees

**Fig 1:**
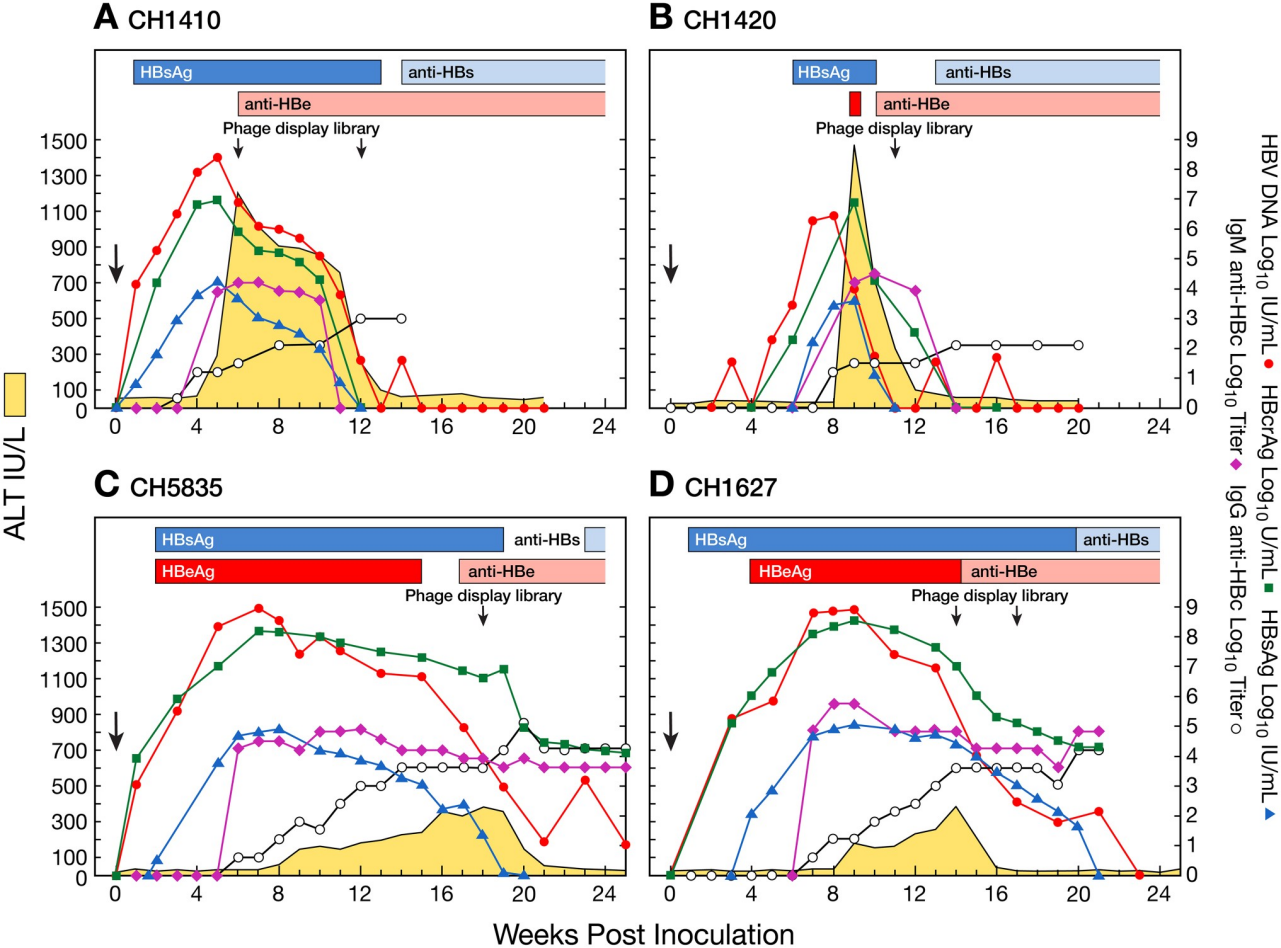
Biochemical, serologic, and virologic course of acute HBV infection in chimpanzees. (A and B) CH1410 and CH1420 were infected with a precore HBV mutant[15] and CH5835 and 1627 (C and D) with the wild-type HBV strain [11, 14]. Yellow areas indicate values for alanine aminotransferase. Each horizontal bar, which is identified by a different color, indicates the time at which serum HBsAg, HBeAg, anti-HBs and anti-HBe were positive. Each line, identified by a different color, indicates the serum titer of HBsAg, HBV DNA, HBcrAg, and IgG and IgM anti-HBcAg (logarithmic scale). The arrows indicate the time at which phage display libraries were performed in chimpanzees using liver specimens from different time points during the course of HBV infection.

Two chimpanzees (CH1410 and CH1420) infected with serum containing a precore HBV mutant implicated in fulminant hepatitis B [15] (*adr*, genotype C; 10^−1^ and 10^−7^, respectively), developed an unusually severe AHB characterized by the highest ALT peaks ever seen in experimental studies of HBV transmission to chimpanzees (1202 IU/L for CH1410 and 1468 IU/L for CH1420) [15]. In contrast, the two animals (CH1627 and CH5835) infected with serum containing 10^8^ genome equivalents of wild-type HBV (*ayw*, genotype D) developed a classic AHB with significantly lower ALT values, with similar peaks ranging from 387 IU/L for CH1627 and 384 IU/L for CH5835 [11, 14]. Remarkably, the ALT peak occurred significantly earlier in severe than in classic AHB, i.e., at week 6 for CH1410 and at week 9 for CH1420 compared to 14 and 18 weeks post-infection, respectively, in classic AHB (Fig 1A–1D). Notably, in severe AHB, HBsAg became detectable at week 2 and 6, respectively, post-infection, but in both animals lasted for a time significantly shorter compared to that in classic acute hepatitis B. Moreover, the titer of HBsAg was an order of magnitude lower (3.88 Log10 IU/mL, ±0.34 SEM) than those seen in classic acute hepatitis B (4.97 Log10 IU/mL, ±0.07 SEM). Seroconversion to anti-HBs occurred at weeks 14 and 13, respectively, in CH1410 and in CH1420, thus more than 10 weeks earlier compared to classic AHB, in which it occurred at week 21 and 23, respectively (Fig 1A–1D). There was no evidence of HBeAg in CH1410 and only a single positive sample in CH1420, whereas in both animals with classic AHB, HBeAg lasted for 10 and 12 weeks, in CH5835 and CH1627, respectively. In both severe and classic AHB, HBV DNA peaked between week 4 and 8 post-infection, with similarly high values of HBV DNA reaching 8.9 Log_10_ IU/mL in all animals except for CH1420 (6.5 Log_10_ IU/mL) that was infected with a diluted inoculum (10^−7^) of the precore HBV mutant. However, despite the lower titer of HBV DNA in CH1420, the interval between the HBV DNA peak and the ALT peak was significantly shorter in both animals with severe AHB (1 week in both animals before the respective ALT peak), regardless of the levels of HBV DNA, compared to classic AHB (5 and 11 weeks before the respective ALT peak) as illustrated in Fig 1A–1D. Serum HBV DNA decreased after the ALT peak in all animals, although the decline was more rapid and sharp in severe than in classic AHB. HBV DNA became undetectable in severe AHB several weeks before classic AHB, concomitant with anti-HBs seroconversion, which occurred much earlier in severe AHB. The pattern and levels of hepatitis B core-related antigen (HBcrAg) paralleled that of HBV DNA in all animals (Fig 1A–1D), indicating that HBcrAg is a sensitive marker of HBV DNA replication. As seen for HBV DNA, in severe AHB, HBcrAg became undetectable several weeks earlier than in classic AHB, where HBcrAg persisted positive, despite at lower levels, throughout the observation period, for at least 5 weeks after the clearance of HBsAg and antibody seroconversion.

### Liver pathology and immunohistochemistry

**Fig 2:**
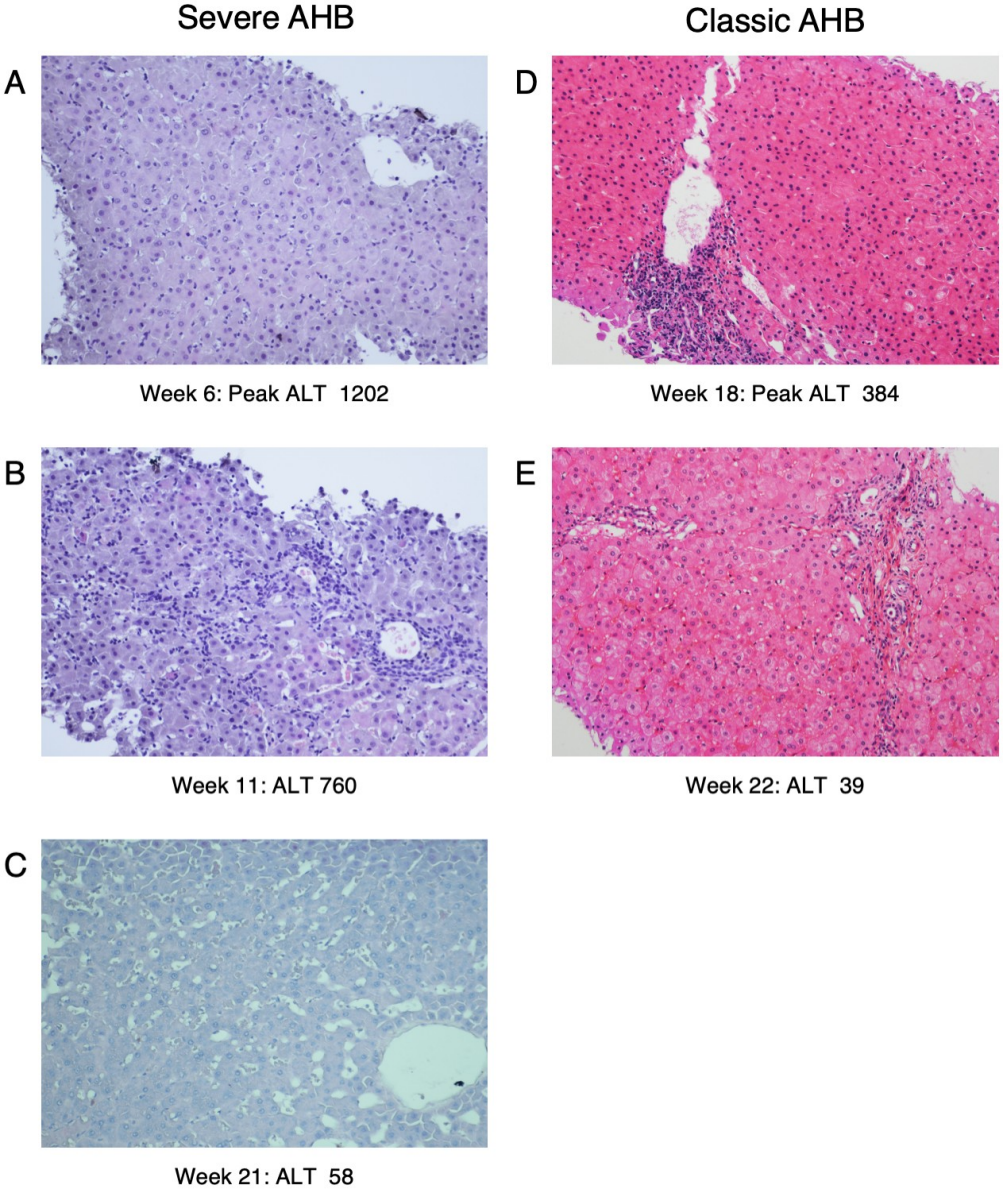
Comparison of the histopathologic evaluation of representative chimpanzees with severe and classic acute hepatitis B (AHB) at different time points. (A, B, C) Histopathologic changes during the course of severe AHB in CH1410 inoculated with a precore HBV mutant implicated in ALF in humans. At the time of the ALT peak (1202 IU/L) at week 6 there was an extensive hepatocellular damage with the presence of hydropic swelling and acidophilic bodies [16] in the absence of inflammatory response (A), which appeared later at week 11 (B), followed by complete resolution (C). (D and E) Histopathologic changes during the course of classic AHB in CH5835 inoculated with wild-type HBV. In this animal the ALT peak (384 IU/L) at week 18 coincided with extensive necroinflammatory changes, followed by resolution of the disease (hematoxylin and eosin, x20).

Analysis of serial liver biopsies from CH1410 with severe AHB showed evidence of generalized hepatocyte damage at the time of the ALT peak (week 6, ALT 1202) (Fig 2A; S1A and S1B Fig), which was more prominent in the central area, consistent with a cytopathic effect (hydropic swelling and acidophilic bodies) [16], but very limited evidence of inflammatory response, which instead appeared later with necroinflammatory changes that peaked at week 11 (Fig 2A–2C and S1B Fig). In contrast, biopsies from CH5835 with classic AHB showed the highest degree of inflammation in the biopsy taken at the time of the ALT peak (week 18) (Fig 2D and 2E) [10]. When we compared the severity of the disease, we observed that the necroinflammatory reaction was more severe in the chimpanzee infected with the precore HBV mutant than that with the wild-type (Figs 1 and 2A–2E). A comprehensive analysis of the cytokines in serial serum samples obtained from the two chimpanzees with severe AHB and the two with classic AHB is under way in our Lab to investigate whether the cytokines profiles differ between the 2 groups (Engle et al, in preparation).

**Fig 3:**
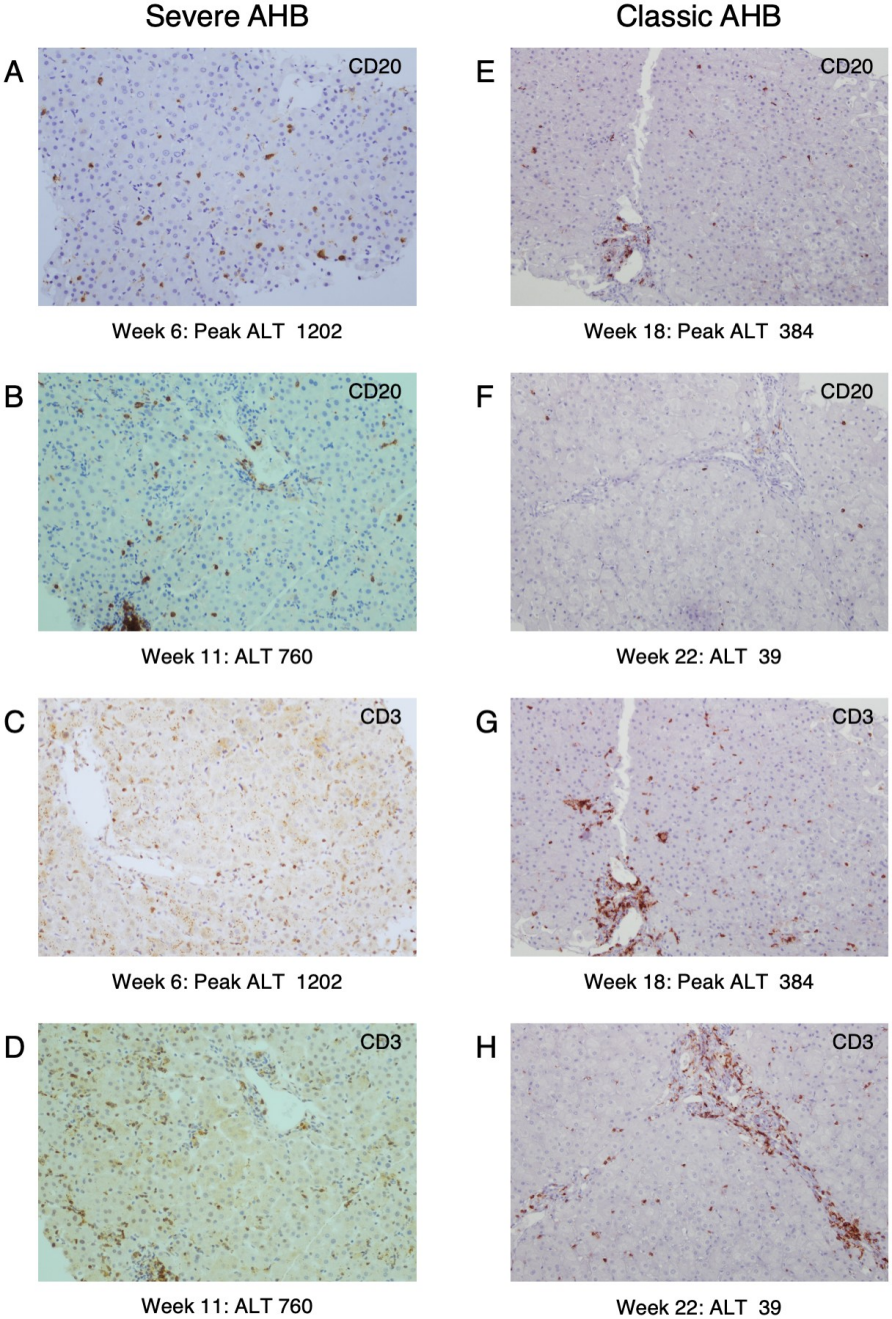
Comparison of immunohistochemical staining of B-cell and T-cell lineages in liver sections of representative chimpanzees with severe and classic acute hepatitis B (AHB) at different time points. Liver sections were stained with antibodies specific for B cells (CD20) (A and B) and T cells (CD3) (E and F). Infiltration of B cells, as single cells (A) or organized in clusters in the portal areas (B), was more extensive in severe than in classic AHB (E and F). In CH1410 with severe AHB (C), T cells at the time of the ALT peak were distributed as single cells within the lobule whereas in classic AHB (G), T cells were more abundant and were present both as single cells in the lobule and as clusters in the portal areas. T cells became more numerous after the ALT peak in severe AHB (D) and remained detectable during the resolution phase in classic AHB (H). The images of CH5835 regarding the histopathology, CD20 and CD3 staining were previously reported [10].

**Fig 4:**
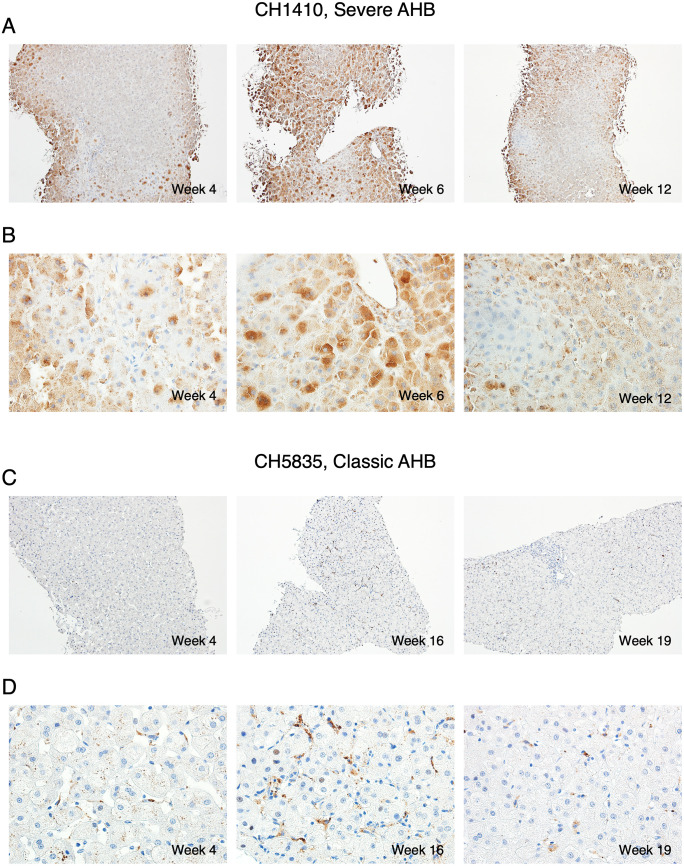
Immunohistochemical staining for caspase 3 activation in serial liver biopsies during acute HBV infection in chimpanzees. (A and B) Paraffin sections from CH1410, infected with a precore HBV mutant [15], and CH5835 (C and D), infected with the wild-type HBV strain [11, 14]. Immunostaining showed the highest caspase 3 activation in CH1410 (A and B) at the time of the ALT peak (week 6, ALT 1202). Panel A, 10X magnification images and panel B, 40X magnification images. (C and D) Immunostaining in CH5835 did not show detectable caspase 3 activation throughout the course of the disease. Panel C, 10X magnification images and panel D, 40X magnification images. AHB denotes acute hepatitis B.

Immunohistochemical staining in severe AHB showed that at week 6 there was an infiltration of CD20-positive B cells distributed as single cells within the lobule, which increased at week 11, after the ALT peak, when they also appeared as aggregates in the portal areas (Fig 3A and 3B), whilst in classic AHB, at the time of the ALT peak, they were rarely detected within the lobule and appeared as small aggregates in the portal tracts (Fig 3E and 3F) [10]. In severe AHB, we observed some plasma cells positive for Mum-4 and cytoplasmic IgM (S1B Fig). At week 6 the liver of severe AHB was also infiltrated by CD3-positive T cells distributed as single cells within the lobules (Fig 3C and 3D and S1B Fig). However, at variance with classic AHB (Fig 3G and 3H), the number of T cells was lower at the time of the ALT peak to increase thereafter (week 11), when they also appeared to be distributed in clusters in the portal areas (Fig 3C and 3D). The vast majority of the T cells were CD8 positive (S1B Fig). Immunostaining for caspase 3 activation, in serial liver biopsies from CH1410 with severe AHB showed that the highest expression correlated with the time of the ALT peak (week 6, ALT 1202) (Fig 4A and 4B). In contrast, CH5835 did not show detectable caspase 3 activation throughout the study (Fig 4C and 4D), suggesting a different mechanism of hepatocyte death. Thus, our data showed a dramatic difference in caspase 3 activation between chimpanzees infected with the precore mutant or the wild-type HBV.

### Isolation and characterization of anti-HBcAg antibodies from the liver of chimpanzees with severe AHB

**Fig 5:**
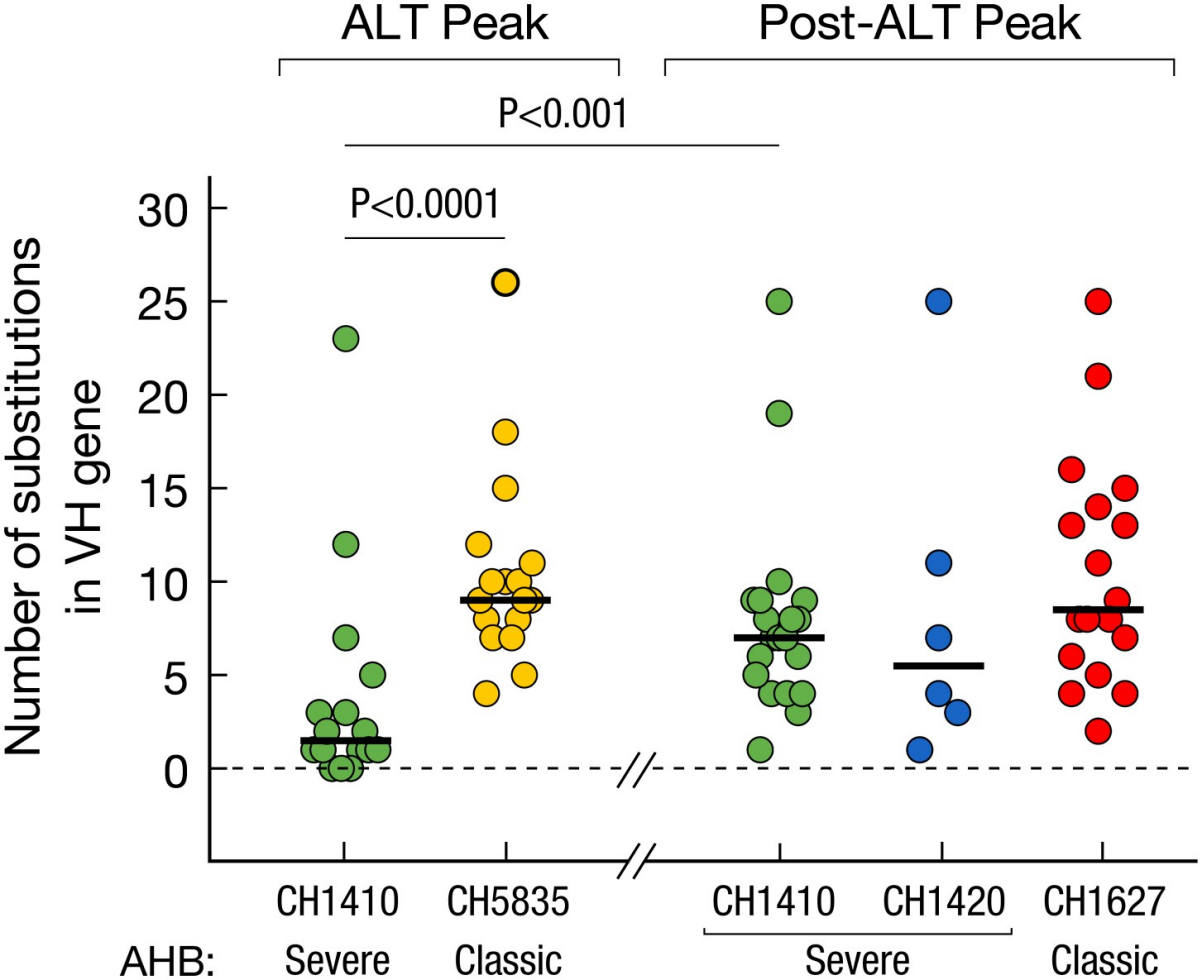
Average frequency of somatic hypermutation in VH genes coding for anti-HBc antibodies among IgG sequences obtained from the liver of chimpanzees with severe and classic AHB. Solid lines show the medians. *P* values refer to comparisons performed using the two-tailed Mann-Whitney test.

RT-PCR amplification of intrahepatic antibody heavy chain genes with primers used for generating human phage display libraries [10] failed to detect IgM heavy chain genes in the two chimpanzees with severe AHB (CH1410 and CH1420), suggesting the absence or very limited presence of IgM-producing B cells in the liver of these animals. In contrast, IgG heavy chain genes were readily amplified by RT-PCR. Panning of IgG phage display libraries against homologous HBcAg cloned and expressed from the same chimpanzees identified several HBcAg-specific antibodies in both animals with severe AHB. The early liver sample from CH1410, obtained at the time of the ALT peak (week 6), showed a remarkably low frequency of somatic hypermutations (SHM) in VH genes coding for HBcAg-specific antibodies (Fig 5), which was similar to that seen in the liver of patients with HBV-associated ALF whose intrahepatic antibody repertoires were dominated by germline, unmutated anti-HBcAg antibodies [10]. In contrast, when we measured the rate of SHM in classic AHB at the time of the ALT peak, we found a significantly higher mutation rate in classic (CH5835) than in severe AHB (CH1410) (Fig 5). However, a significantly higher number of SHM was also seen in the same animal with severe AHB (CH1410) when the sample was collected at a later stage of disease (6 weeks post-ALT peak), indicating that SHM emerged over time. This finding was confirmed in the other chimpanzee (CH1420) with severe AHB, in which the earliest available liver sample was obtained after the ALT peak (at week 12), which was sharp, with levels of ALT rising from 20 to 1468 IU/mL in one week. Thus, our data showed that the frequency of SHM significantly differed between severe and classic AHB (Fig 5) during the early stage of the disease, but not after the ALT peak.

Phage display libraries identified several intrahepatic HBcAg-specific antibodies in germline configuration that were seen only in severe AHB but not in classic AHB. Thus, the pattern observed in severe AHB was reminiscent of that documented in the liver of patients with HBV-associated ALF who underwent liver transplantation within one week after the onset of symptoms. However, while in the liver of HBV ALF patients we found a high prevalence of both IgG and IgM against HBcAg, in severe and classic AHB in chimpanzees only IgG anti-HBcAg antibodies were cloned from the livers tissue. Altogether, these data point to a close similarity between the early stage of severe AHB caused by a precore mutant virus in chimpanzees and ALF in humans, which is also strongly associated with precore mutant HBV strains, although the two conditions diverge in the subsequent disease evolution, with ALF resulting in massive hepatic necrosis that is often fatal while severe AHB evolves toward a clinical and pathological picture that eventually turns into that of classic AHB.

**Fig 6:**
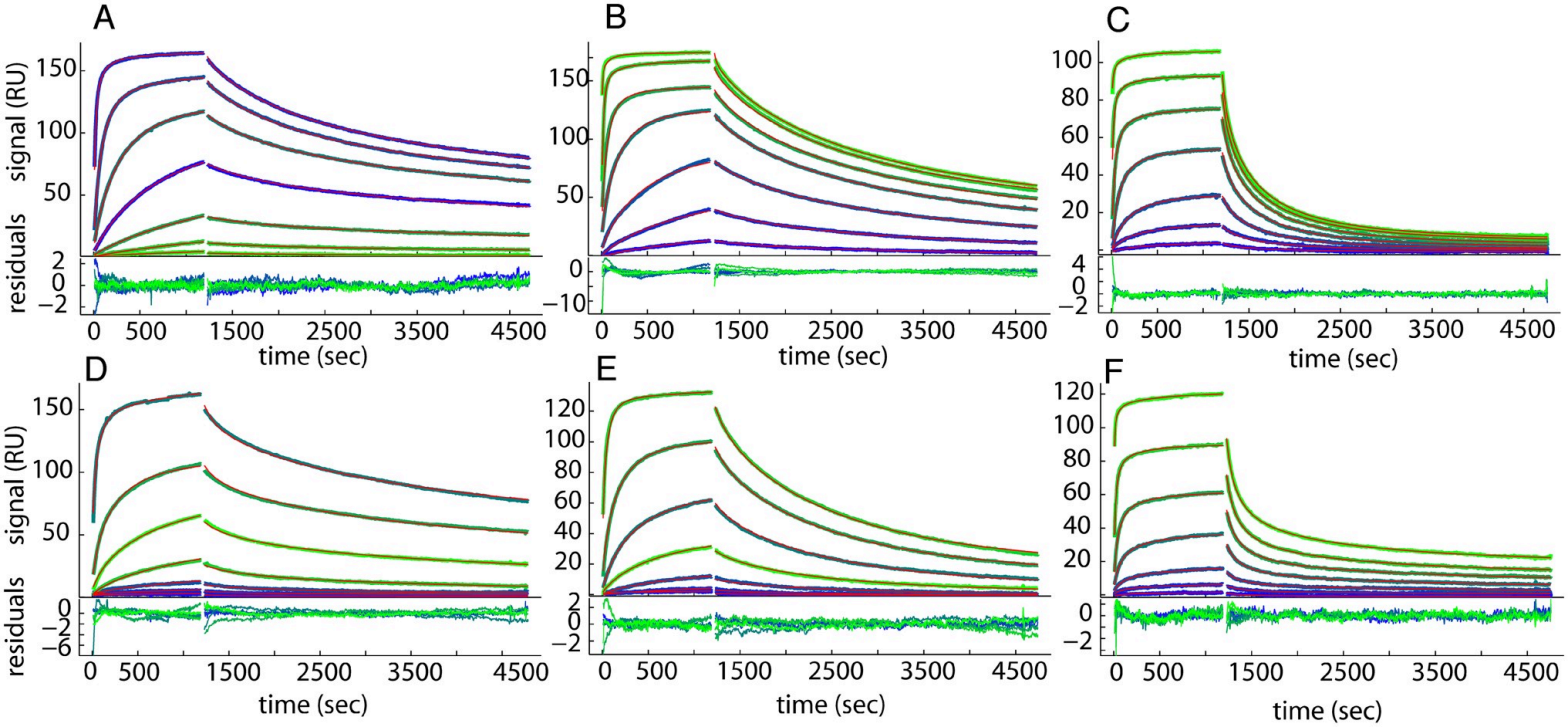
Representative sensograms showing binding of monoclonal antibodies (Fab) to HT core immobilized on the CM5 sensor surface. SPR binding data (blue/green lines: 0.3, 1, 3, 10, 30, 100 and 500 nM) and best-fit (red lines, superimposed by the blue/green lines) from the surface site distribution model in EVILFIT. For each panel, the binding traces and best-fit are shown on the top and the residuals of the fits are shown on the bottom. (A) Fab B6, (B) Fab E6, (C) Fab B2, (D) Fab D5, (E) Fab G4, (F) Fab C7. In spite of the narrow range of K_d_ values as shown in Table 1, the binding kinetics appear to be distinctive as shown from dissociation phase (1200–4800 seconds), with Fab B2 and Fab C7 showing faster dissociation than the other Fab molecules.

The affinity for the homologous HBcAg of 6 anti-HBcAg Fabs with no or few SHM isolated by phage-display libraries from the liver of CH1410 with severe AHB at the time of the ALT peak was measured by surface plasmon resonance (SPR) (Table 1 and Fig 6). Despite having no or only a single mutation in the VH gene, these Fabs had high binding affinities to the homologous HBcAg, with Kd values between 1 and 36 nM. These affinities are higher than those reported for unmutated germline-like antibodies [17], but lower than those of germline antibodies isolated from human ALF, which were in the picomolar to subnanomolar range [10].

**Table 1:** Binding affinities of selected anti-core Fabs in germline configuration.

Fab	VH genes			Mutation*	K_d_(nM)
	V	D	J		
B6	V180-RF-AACZ04069749.1	D5-12*01	J6*03	1	1.0
E6	V171-RF-AACZ04069747.1	D5-12*01	J6*03	1	1.09
B2	V171-RF-AACZ04069747.1	D1-14*01	J6*03	1	6.79
D5	V80-RF-AACZ04001323.1	D5-12*01	J4*02	0	7.00
G4	V173-RF-AACZ04069748.1	D6-19*01	J5*02	0	17.8
C7	V75-RF-AACZ04001323.1	D3-9*01	J4*02	1	36.4

* Number of amino acid substitutions in VH gene.

### Characteristic of liver VH repertoires in chimpanzees with severe AHB

**Fig 7:**
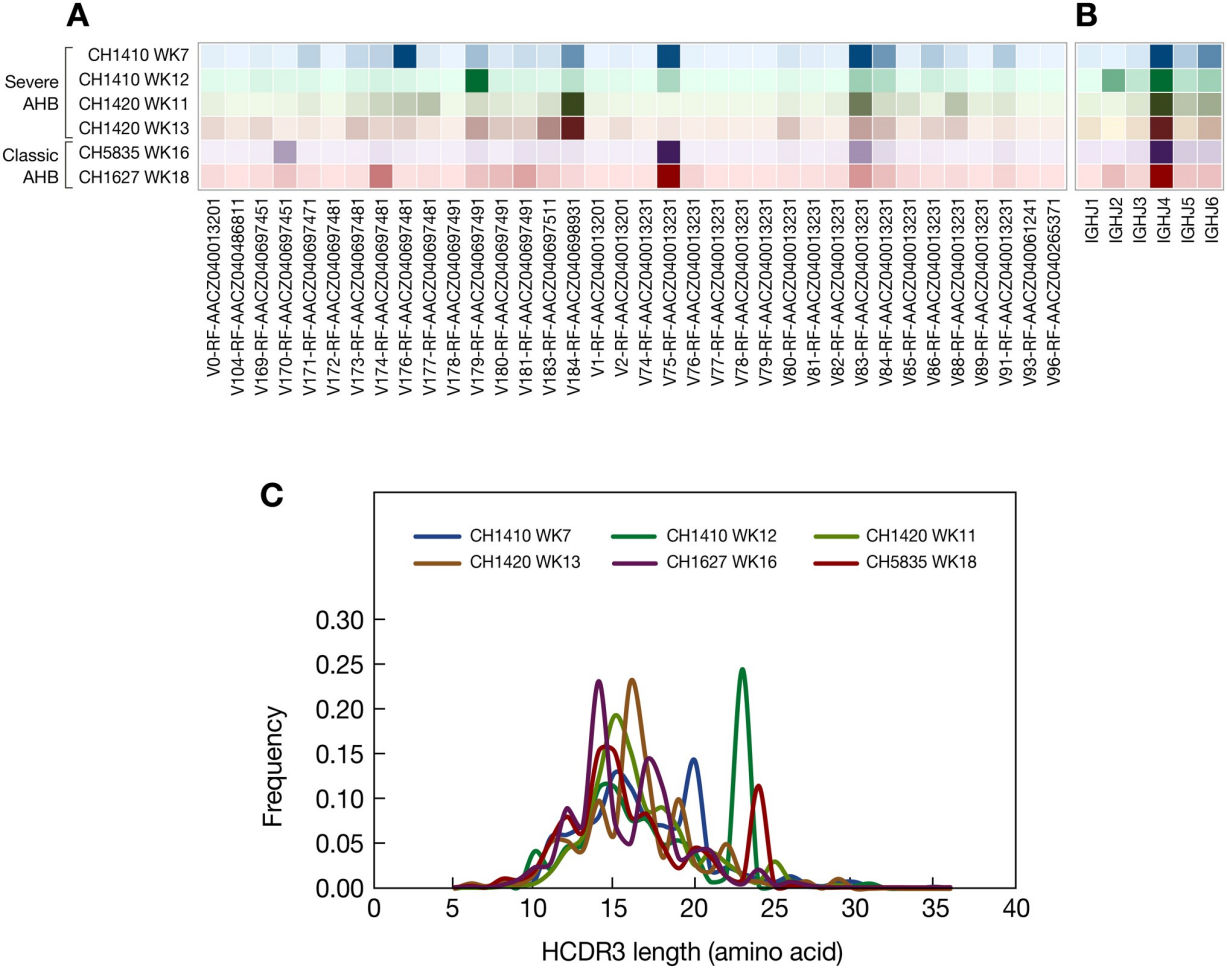
Comparison of the antibody repertoires in the livers of four chimpanzees between severe and classic AHB at different time points during the course of HBV infection. (A) V gene usage by individual chimpanzee. (B) J gene usage by individual chimpanzee. Increased color intensity indicates higher frequency of gene usage. Chimpanzees are colored as in panel C. (C) HCDR3 length distribution of IgG for each chimpanzee. CDRH3 lengths were determined using the Immunogenetics (IMGT) numbering scheme.

To further characterize the intrahepatic antibodies in chimpanzees with severe AHB, the entire liver VH repertoire was analyzed by next-generation sequencing (NGS) (Table 2). The analysis was conducted on two time points from two animals with severe AHB and one time point from two animals with classic AHB, according to the availability of liver specimens. As shown in Fig 7A, each repertoire was composed of diverse VH genes with a distinct distribution of VH gene usage. However, no clear difference in VH gene usage was found between severe and classic AHB. As reported for human VH repertoires [18], the IGHJ4 gene was the most abundantly used J gene across the 6 repertoires (Fig 7B). The HCDR3 length was found to be in a range between 10 and 20 aa, with a peak of 15 aa. Three repertoires also had a higher second peak in the samples taken after the ALT peak: CH5835, week 18, at 20 aa; CH1410, week 12, at 23 aa; and CH1627, week 16, at 24 aa (Fig 7C). Interestingly, we also found substantial VH genes sharing by different chimpanzees, reflecting a repertoire overlap between different individuals, as was previously observed in humans (S2 Fig) [18].

**Table 2:** Sequence metadata.

IgG Repertoire	Raw reads	Duplicated sequence	Unique sequence	Clones
**CH1410_WK7**	46,515,617	25,005,658	995,426	83,631
**CH1410_WK12**	43,264,437	24,224,855	830,491	55,274
**CH1420_WK11**	44,724,189	21,038,662	816,968	68,662
**CH1420_WK13**	41,922,442	12,833,486	522,080	39,912
**CH1627_WK16**	41,636,263	20,800,028	797,391	53,920
**CH5835_WK18**	42,169,176	31,417,481	859,194	41,629

**Fig 8:**
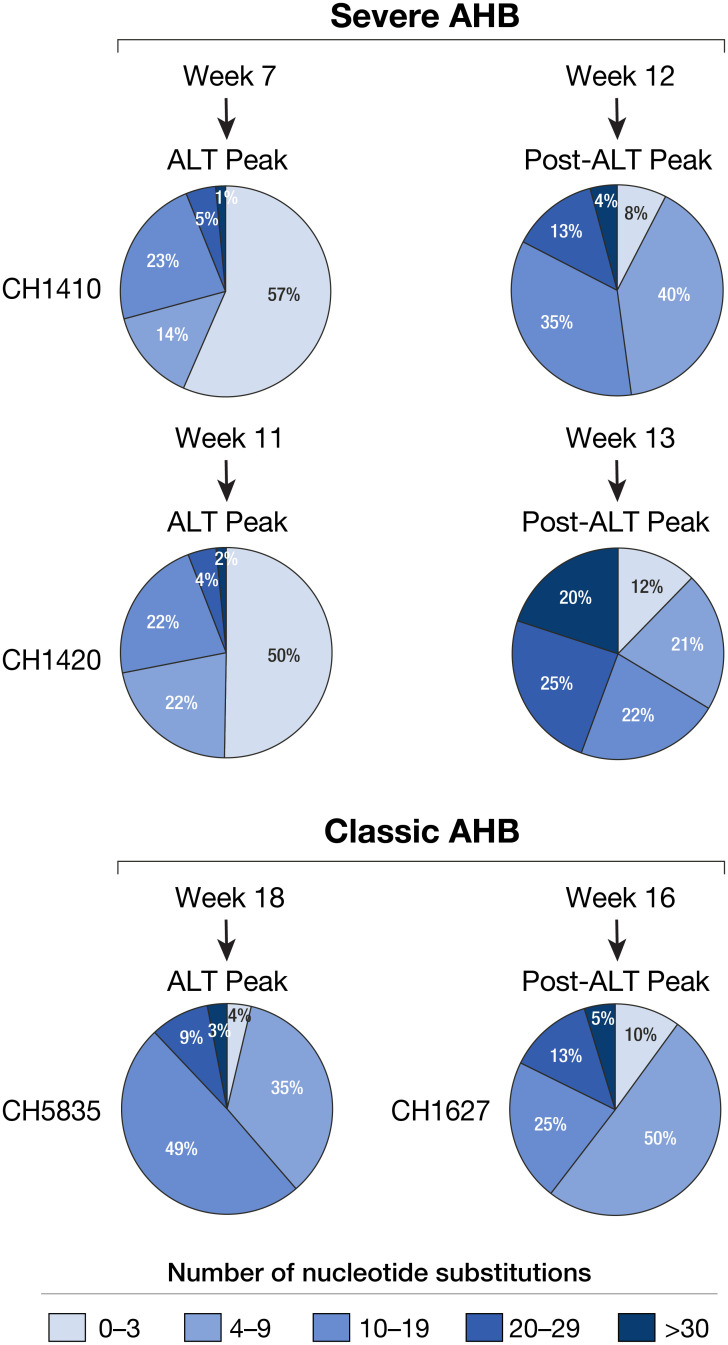
V gene somatic mutations in antibody (VH of IgG) repertoires from the livers of four chimpanzees with either severe or classic AHB at different time points during the course of HBV infection. V genes with 0–3 nucleotide substitutions are defined as unmutated antibodies in germline configuration. Numbers of VH genes in each mutation category, calculated as percentage of total VH genes analyzed, are shown in different shades of blue within the pies.

The level of SHM in VH genes from different repertoires was markedly different. The level of SHM increased with the disease progression, as seen in CH1410 from week 7 to week 12 and in CH1420 from week 11 to week 13. When severe and classic AHB were compared, the VH genes from severe AHB carried a significantly lower number of SHM than those from classic AHB at the same disease stage, as illustrated by comparison between CH1410 at week 7 and CH5835 at week18, as well as after the ALT peak, between CH1420 at week 11 and CH1627 at week 16 (Fig 8). Importantly, all the anti-HBcAg antibody genes identified in the phage-display libraries were not only found in the corresponding repertoires, but in most cases were highly expanded (S1 Table), which contrasts with the findings in patients with ALF where anti-HBcAg antibody genes were found in the respective repertoires, but no clonal expansion was documented [10].

## Discussion

The objective of the present study was to investigate the pathogenesis of severe AHB caused by a precore HBV mutant and compare it with that of classic AHB caused by wild-type HBV, using archived serum and liver samples from historical chimpanzees studies [11, 14, 15]. Our results shed new insights into the relationship among biochemical markers of liver damage, serological and viral kinetics, and immunological determinants in severe versus classic AHB. Despite the inherent limitations of using historical chimpanzee studies, the availability of serial serum and liver specimens collected longitudinally for at least 6 months throughout the course of acute HBV infection allowed us to identify distinct-outcome specific factors that differentiate severe from classic AHB. First, severe AHB was characterized by a significantly shorter incubation period, an early and remarkable ALT peak, and an unusually rapid viral clearance associated with accelerated antibody seroconversion. All of these features resemble those typically associated with HBV ALF in humans rather than those of classic AHB. Strikingly, the ALT peak not only occurred very early but also within one week from the HBV DNA peak, whereas in classic AHB this interval ranged from 5 to 11 weeks. Second, severe AHB was characterized by the lack or transient detection (in one animal for only one week) of HBeAg, whereas in classic AHB HBeAg persisted in both animals for up to 12 weeks. The biologic function of HBeAg remains largely unknown [19]. HBeAg is not essential for viral replication [20] and has been suggested to exert an immunoregulatory function by inducing tolerance and promoting Th_2_ responses, which may favor HBV persistence [21]. Clinical studies have documented that acute infection with HBeAg-negative mutants may lead to a more severe acute hepatitis and even ALF [6, 22, 23]. Third, the kinetics and magnitude of serum HBV DNA did not differentiate the two disease forms, being similarly high in both severe and classic AHB. Fourth, by analyzing weekly samples during the entire course of AHB from the incubation period to the resolution phase, our study provides conclusive evidence that HBcrAg is a sensitive marker of viral replication since it followed the same pattern as that of HBV DNA, consistent with previous studies performed in the setting of chronic HBV infection [24]. Recent studies have shown that HBcrAg correlates with HBV covalently closed circular (ccc) DNA, which is the template for pregenomic RNA transcription and HBV genome replication [24–26]. Further studies are in progress to investigate the relationship between the levels of HBcrAg and the covalently closed circular DNA (cccDNA) reservoir in the liver, since an unknown proportion of individuals who recover from acute HBV infection still maintain low levels of intrahepatic cccDNA, which may be responsible for HBV reactivation in subjects undergoing immunosuppressive therapy for other disease conditions [27–29]. Whether there is a difference in the levels of the cccDNA reservoir between AHB caused by a precore mutant or the wild-type HBV, remains to be established.

In contrast with classic AHB, in which the liver damage is believed to be T-cell mediated [14, 30, 31], the pathogenesis of HBV ALF still remains to be elucidated in part because of the rapid and dramatic clinical course of this disease, which has posed limitations for pathogenesis studies. We recently found an unexpected overwhelming B-cell gene signature in the liver of patients with HBV ALF, with extensive intrahepatic production of IgG and IgM antibodies in germline configuration directed against the HBcAg and showing a remarkably high binding affinity, comparable to that of potent affinity-matured antibodies, suggesting that HBV ALF is associated with a strong T-cell independent intrahepatic B-cell response. In light of these results, we embarked in this study to investigate whether chimpanzees infected with a precore HBV mutant implicated in human ALF would provide new insights on ALF pathogenesis. Although none of the chimpanzees developed ALF, our data suggest that there is a gradient in disease severity and immunological features in acute HBV disease in chimpanzees, with severe AHB being more closely related to ALF than to the classic form of AHB. These findings raise the important question of whether the pathogenesis of liver damage caused by the precore HBV mutant differs from that of the wild-type virus. We found several distinctive features of the host immune response in severe compared to classic AHB, which were reminiscent of HBV ALF: (*i*) an intrahepatic infiltration of B cells, albeit to a lesser extent than in human ALF, but more abundant than in classic AHB; (*ii*) the presence of intrahepatic anti-HBcAg IgG antibodies in germline configuration or with very low numbers of SHM, which was the hallmark of ALF in humans [10], albeit only in the early phase of severe AHB; (*iii*) the moderate to high affinity binding of selected anti-HBc IgG Fabs in germline configuration that were cloned from chimpanzees with severe AHB, albeit lower than that in ALF where the Fabs affinities were in the nanomolar to picomolar range. Thus, all these features were reminiscent of those of ALF but overall less marked and predominantly associated with the early stages of the disease.

Another major difference between chimpanzees with severe AHB and humans with a hyper-fulminant course of ALF, which led to liver transplantation within one week from the clinical onset, was that no IgM genes were amplified by RT-PCR from the liver of chimpanzees. The lack of intrahepatic IgM anti-HBcAg, the lower mutation rate of IgG VH genes and the lower binding affinities of germline anti-HBcAg antibodies in chimpanzees with severe AHB compared to ALF may partially explain the failure of this disease to run a fulminant course, eventually switching toward a more classic clinical outcome. Interestingly, when we compared the rate of SHM in severe versus classic AHB at the same disease stage (at the peak of ALT level), we found a significantly lower mutation rate in VH genes from CH1410 with severe AHB than in CH5835 with classic AHB, which may suggest that T-cell-induced SHM is delayed in chimpanzees with severe AHB. Whether and by what mechanism a delayed T-helper activity may contribute to pathogenesis remains to be elucidated.

One of the strengths of this study is that the results obtained by phage-display libraries were validated by NGS analysis on liver immunoglobulin VH repertoires. The data were consistent with those of anti-HBcAg library selection demonstrating a significantly lower extent of SHM in VH genes from severe AHB than from classic AHB at the same disease stages. Likewise, the SHM rate in VH genes increased at the later disease stage in chimpanzees with severe AHB. Altogether, these data further suggested that the T-cell help in chimpanzees with severe AHB eventually occurred but was temporally delayed. Consistent with this concept, we have also noticed that all the anti-HBcAg antibody-encoding genes were not only found in the corresponding repertoires in both forms of AHB, but most of the clones were activated and expanded, a finding that was not observed in human ALF. This result reinforces the notion that T cells are functional in chimpanzees with both severe AHB and classic AHB, unlike those of patients with ALF that seem to be stunned in a non-functional, anergic state, associated with the expression of negative regulators of T-cell activation such as CTLA-4 [10].

Although infected chimpanzees developed severe AHB characterized by a rapid disease onset and high ALT levels, the infection eventually switched toward a self-limited form, similar to that of classic AHB, and the chimpanzees completely recovered. The reasons why an ALF-associated HBV strain did not trigger a fulminant course in chimpanzees as it did in humans remain unclear. Consistent with the generally lower pathogenicity of human hepatitis virus infections in the chimpanzee model [32], it is likely that non-human primates possess inherent immunologic or genetic factors that make them less susceptible to the disease mechanisms that cause ominous consequences in humans or that cross-species barriers exist despite the very close similarity between humans and chimpanzees at the genomic level.

We still do not know why chimpanzees infected with an HBV strain that caused ALF in humans resulted in higher ALT levels than in classic acute HBV infection caused by wild-type HBV. Since the wild-type HBV virus belongs to genotype D whereas the precore mutant was from genotype C, one could speculate that different genotypes may manifest different virulence in chimpanzees. However, an earlier study by Barker et al. did not find any difference in virulence of different HBV genotypes in chimpanzees [33]. Based on our data, which showed that the ALT peak was associated with extensive liver damage and caspase 3 activation in the presence of a very limited inflammatory reaction, an intriguing possibility is that precore HBV mutants have direct cytopathic activity and therefore may cause an early hepatocytolysis without the intervention of immune-mediated mechanisms. Indeed, preliminary data from our laboratory showed that classic AHB in chimpanzees, but not severe AHB, correlated with an early and robust induction of cytokines in serum involved in T-cell responses, in agreement with the model of T-cell-mediated pathogenesis and viral clearance (Engle et al. in preparation). The lack of induction of cytokines associated with adaptive T-cell responses in severe AHB argues against a significant role of classic T cell-mediated immune responses in the pathogenesis of this severe form of hepatitis in the chimpanzee model. In addition, the genotype-C HBV strain that was extensively characterized by Ogata et al. [15] and the genotype-D HBV strains that were recently sequenced using NGS by Chen et al. [10] from patients with ALF shared numerous mutations both within and outside the HBcAg, besides the precore stop codon at nucleotide 1896, making it difficult to identify the phenotype associated with specific mutations in ALF.

In conclusion, although the number of chimpanzees included in this study was limited, access to archived serial serum and liver specimens from four chimpanzees allowed us to identify distinct outcome-specific factors that differentiated severe from classic AHB. In particular, our data demonstrated that severe AHB was characterized by a shorter incubation period, an early and remarkable ALT peak, an unusually rapid viral clearance, and an accelerated antibody seroconversion, along with antibodies in germline configuration albeit only in the early phase of severe AHB, which altogether bear closer resemblance to HBV ALF than to the classic form of AHB.

## Methods

### Study subjects

Chimpanzees. To compare severe AHB with classic AHB, we studied archival weekly serum and liver biopsy specimens from 4 chimpanzees followed for at least 6 months after inoculation that were included in previous studies aimed at investigating the pathogenesis of acute hepatitis B. Two animals (CH1410 and CH1420) were inoculated with a precore HBV mutant implicated in fulminant hepatitis B in humans [15], whereas the other two (CH5835 and CH1627) were inoculated with the wild-type HBV strain (ayw) [11, 14]. Chimpanzees inoculated with the precore mutant received a 10^−1^ (CH1410) and 10^−7^ (CH1420) dilutions, respectively, of the index serum which had a titer estimated to be 8.0 log_10_ copies/mL. Thus, CH1410 received about 7 log_10_ copies and CH1420 about 1 log_10_ copies, whereas those inoculated with the wild-type (serotype ayw) received 8.3 log_10_ copies/mL. The original precore mutant and wild-type HBV inocula were of similar magnitude. However, CH1420 received a significantly lower amount of virus compared to CH1410. In all animals, serum ALT levels were measured in serial serum samples, obtained at weekly intervals throughout the study.

### Ethics statement

The animals were caged individually and handled according to guidelines specified by law and approved by the Animal Care and Use Committees of the National Institute of Allergy and Infectious Diseases (ILARC (1996) Guide for the Care and Use of Laboratory Animals (National Academies Press, Washington, DC).

### Serologic and virologic kinetics of chimpanzees with severe AHB

Weekly serum samples from all 4 chimpanzees were tested for standard HBV serological markers, as well as for titers of HBsAg, anti-HBs, antibody to HBcAg (anti-HBc), IgM anti-HBc, hepatitis B e antigen (HBeAg), anti-HBe, with commercial enzyme immunoassays (Abbott Laboratories and Diasorin). Titers of IgM and IgG anti-HBc were obtained with enzyme immunoassays by testing 2-fold serial dilutions. HBsAg was quantified, relative to a WHO reference (NIBSC, Potters Bar, Herefordshire, ENG 3QG code:03/262) reagent standard. Samples were diluted to a linear analysis range, tested along with an internal standard control and values were expressed as log_10_ IU/mL. The effective dynamic range was 0.07 to 8.0 log_10_ IU/mL. Levels of serum HBV DNA were determined with an in-house quantitative real-time PCR method described previously [34]. Briefly, **t**otal DNA was purified from serum samples using QIAamp DNA Blood Mini Kit (Qiagen). The primers and probe were located near the 5’ end of the S gene. Each 20 μL reaction contained 45 pmol of forward (5’- GGA CCC CTG CTC GTG TTA CA-3’) and reverse (3’- TTG AGA GAA GTC CAC CAC GAG TC-5’) primers, 12.5 pmol of non-fluorogenic-quenched-probe (6FAM- TGT TGA CAA GAA TCC TCA) and TaqMan Fast Universal PCR Master Mix (Applied Biosystems, Foster City, CA). PCR was performed using an ABI PRISM 7900HT Sequence Detection System (Applied Biosystems). Conditions included incubation at 95°C for 20 seconds, followed by 45 PCR cycles of 1 second at 95°C, and 20 seconds at 60°C. Viral titers were expressed as log_10_ IU per mL. The quantities of HBV DNA were calibrated using the WHO international standard (97/746), as previously described by Saldanha *et al* [35]. Hepatitis B core related antigen (HBcrAg) was assayed with the Lumipulse G HBcrAg kit and a Lumipulse G1200 Analyzer (Fujirebio, Tokyo, Japan). The test has a 4-log dynamic range (3.0 to 7.0 log10 U/mL) and a lower detection limit of 2.6 log10 U/mL. Samples that fell outside the upper limit of detection were diluted and re-tested to obtain results within the linear analysis range [36].

### Liver pathology and immunohistochemistry

Archived weekly paraffin-embedded liver biopsy (FFPE) specimens were available from all the chimpanzees included in this study. However, the quality of the staining of FFPE sections obtained from serial liver biopsies of CH1420 was of poor quality, so the immunohistochemical data obtained from this animal could not be evaluated. FFPE sections were stained with hematoxylin and eosin for histopathological examination by an expert hepatopathologist (S.G.). Formalin- fixed paraffin-embedded liver sections obtained at three time-points during the course of HBV infection were used to perform immunohistochemical staining using a panel of antibodies that included CD3, CD8, CD20, IgG, IgM, Mum-1, C1q, CD4, CD138 (AbD Serotec) and cleaved caspase-3 (Cell Signaling Technology, 9661, Boston, MA). We also used CD8 (Leica Biosystems) and CD163 (Thermo Scientific). Briefly, sections of 3 to 5 μm were deparaffinized through graded alcohols and xylene. Immunohistochemical staining was performed after antigen retrieval using either citrate buffer (10 mmol, pH 6.0) or EDTA (1 mmol, pH 9.0). Slides were incubated in Tris-goat serum (3%) for 15 min and then incubated at room temperature with primary antibodies. Detection was carried out on the automated system BenchMark XT autostainer (Ventana Medical Systems) or Bond RX (Leica Biosystems) platform according to the manufacturer-supplied protocols. The immunohistochemical staining of the two chimpanzees with classic AHB following inoculation with the wild-type HBV (ayw) were previously reported [10].

### Expression and purification of specific HBV core particles

The viral genomic sequence encoding HBcAg of a precore HBV mutant (strain HT) was synthesized by Gene Art (Invitrogen) using the sequence previously published [15]. The gene was cloned into pET14b at NcoI and XhoI sites and confirmed by sequencing. *E*. *coli* cells BL21(DE3) pLysS were transformed with the recombinant plasmids carrying the HBcAg-encoding gene and grown for expression as previously reported [10]. Briefly, a single bacterial colony was inoculated into 10 ml of LB media and incubated with shaking at 37°C overnight. After overnight culture, the cells were transferred into 500 ml of fresh LB and cultured for 2–4 h at 37°C. The expression of HBcAg was induced by addition of IPTG to a final concentration of 0.2 mM and the culture was maintained for an additional 4 hrs. The cells were collected by centrifugation at 5000xg for 15 min, resuspended in 40 ml of 1X PBS, and lysed through three cycles of freeze/thaw. The HBV core particles in the supernatant following centrifugation were precipitated by addition of ammonium sulfate to final 25% saturation and incubation with rotation at 4°C for 2h. The pellet was collected after centrifugation and resuspended in 3 ml of 1X PBS. The HBV core particles were finally purified by ultracentrifugation on 15–45% sucrose gradient, buffer-exchanged and concentrated in 1X PBS by ultrafiltration with a MW cutoff of 100 kD and sterilized by filtration through a 0.22 um filter. The purity and concentration of the HBV core particles were estimated by SDS-PAGE analysis in parallel with a known concentration of commercially available HBV core protein; the identity was confirmed by ELISA using a polyclonal anti-HBc antibody.

### Construction of Fab-display phage libraries

Phage-display libraries were constructed from total RNA extracted from liver tissue of the two chimpanzees with severe AHB (CH1410 and CH1420) at one or two time points during the course of acute HBV infection, as described previously [37]. All the libraries from chimpanzees were IgG1 because PCR amplification of Ig u-chains was negative and therefore no IgM libraries were constructed. Genes coding for Fd [variable (VH) and first constant region (CH1)] and for light chains were amplified by RT-PCR. The cloning of PCR products of heavy and light chain into pComb3H resulted in the generation of phage Fab-display libraries with an average size of 1 × 10^8^ individual clones for each library. The diversity of the unselected library was evaluated by sequencing of 96 randomly selected clones to determine the usage of VH and VL gene segments.

### Library selection

Phage libraries of IgG1 Fab were panned separately by solid-phase selection on ELISA plates coated with homologous HBcAg. Briefly, wells of 96-well plate were coated with 100 μL/well of solution of 5 μg/ml antigen in 1x PBS. Wells were then incubated overnight at 4°C and blocked with 3% milk in 1x PBS. Phage Fab suspensions containing 10^12^ pfu in 100 μL 1x PBS with 2% milk were added and incubated for 2 hours at room temperature. After incubation, phages were aspirated, the wells were washed, and bound phages were eluted, titrated, and replicated as described [38]. After three rounds of panning, 96 randomly picked single phage-Fab clones from each library were screened for specific binding to the respective antigen by phage ELISA as described [37].

### Sequence analysis of anti-HBc Fab antibodies

The heavy variable-region nucleic acid sequences were determined for positive clones in order to establish our cohort of unique anti-HBV mAbs. The closest human germline V(D)J gene segments for each unique sequence were determined using DNAPLOT with IMGT sequence database (http://www.imgt.org/IMGT). The chimpanzee germline VH genes were assigned by comparison of VH genes with entire chimpanzee VH repertoire collected in Vgenerepertoire.org. The framework and CDRs were assigned according to IMGT nomenclature. The somatic mutations were identified by comparison of V-genes (from framework 1 to 3) with the closest germline counterpart. The first 28 bases corresponding to the primer sequence were not included in the calculation. Overall statistical significance in mutation frequency between samples was calculated using two-tailed Mann-Whitney test. *P*-values ≤ 0.05 were considered statistically significant.

### Next-generation sequencing of intrahepatic IgG repertoire and bioinformatics analysis of Illumina paired-end sequencing

To amplify genes coding for Fd (VH + CH1) we used the same set of 5’-end VH family specific primers and 3’-end IgG1-specific CH1 primer that we previously used in the construction of the phage display library, as reported [10]. A total of 6 amplicons were generated, including IgG from liver RNA extracted from two time points of two chimpanzees with severe acute hepatitis, as well as from one time point of two chimpanzees with classic acute hepatitis. The amplicons were purified and subjected to Illumina 2X300 bp paired-end sequencing and analyzed, as described [10]. Next-generation sequencing data have been deposited in the NCBI Sequence Read Archive (SRA) (accession number PRJNA422423).

Paired-end Illumina sequencing reads were merged using FLASH (Fast Length Adjustment of SHort reads) [39]. Primer sequences were removed using FLEXBAR with a 0.3 threshod [40]. The Stand-alone IGBLAST [41] was used for V(D)J germline gene assignments and an in-house developed Antibodyomics1 python script was applied to process IGBLAST outputs [42]. The chimpanzee’s V germline gene was obtained from Dr. David Olivieri [43], and IMGT human D and J germline gene were used for D and J gene assignment. Non-Ig reads, non-duplicate and non-production reads were filtered out. Reads containing stop codon and Phred scores of less than 20 occurring over 80% of the V(D)J region were also discarded from all NGS samples. Only sequences assigned FR1-FR4 were retained in the data set. Unique antibody sequences were extracted from reads passing the above filters and were subjected to further analysis. Only sequences assigned FR1-FR4 were retained in the data set. Unique antibody sequences were extracted from reads passing the steps above and were subjected to further analysis. Unique sequences were parsed into clones, which means that antibodies within the same clone had identical amino acids on HCDR3 and use the same V, J germline gene [44]. Antibody sequences with equal to or fewer than 3 mutations were considered to be in germline configuration. The first nucleotide to the second conserved cysteine codon of the V region was used to evaluate somatic hypermutations. Here, we defined an antibody clone as an antibody that had 100% identical amino acid sequences in the CDR3 and used identical V and J germline genes.

## Supporting information

S1 Fig Clinical and histopathologic course of severe AHB in CH1410 inoculated with a precore HBV mutant implicated in acute liver failure in humans. (A) Clinical, serologic and virologic course of severe AHB. (B) Hematoxylin and eosin (H&E) demonstrated extensive hepatocellular damage with hydropic swelling and acidophilic bodies when the ALT peaked at week 6, followed by the highest degree of necroinflammation after the ALT peak, at week 11. Immunohistochemical staining of T-cell and B-cell lineages in liver tissue at different time points during the course of HBV infection. Liver sections were stained with antibodies against CD3, CD8, CD20, Mum1/IRF4, and IgM. Images of liver sections (week 2 CD3 40X, all others 20X) show an infiltration by B and T cells initially distributed as single cells within the lobule at the time of the ALT peak, followed by an increase after the ALT peak with cells appearing both as single cells as well as clusters within the portal areas. There were also plasma cells positive for Mum1/IRF4 especially after the ALT peak along with rare plasma cells positive for IgM. Staining for IgM was predominantly confined to the sinusoids.
(PDF)

S2 Fig Shared VH gene sequences amongst the four chimpanzees. Numbers shown within circles are the total number of VH genes analyzed. Numbers shown in the overlapping area between circles represent shared sequences between chimpanzees.
(PDF)

S1 Table Expansion of anti-core antibody clones (>100 antibodies). All the anti-HBcAg antibody genes identified in the phage-display libraries were found in the corresponding liver antibody repertoire analyzed by next-generation sequencing, and in most cases were highly expanded.
(PDF)
