# Supplementary figures and images for: Distinct disease features in chimpanzees infected with a precore HBV mutant associated with acute liver failure in humans
Source: PLoS Pathog. 2020 Aug 31;16(8):e1008793. doi: 10.1371/journal.ppat.1008793 (PMC7485984; doi:10.1371/journal.ppat.1008793)

S1 Fig

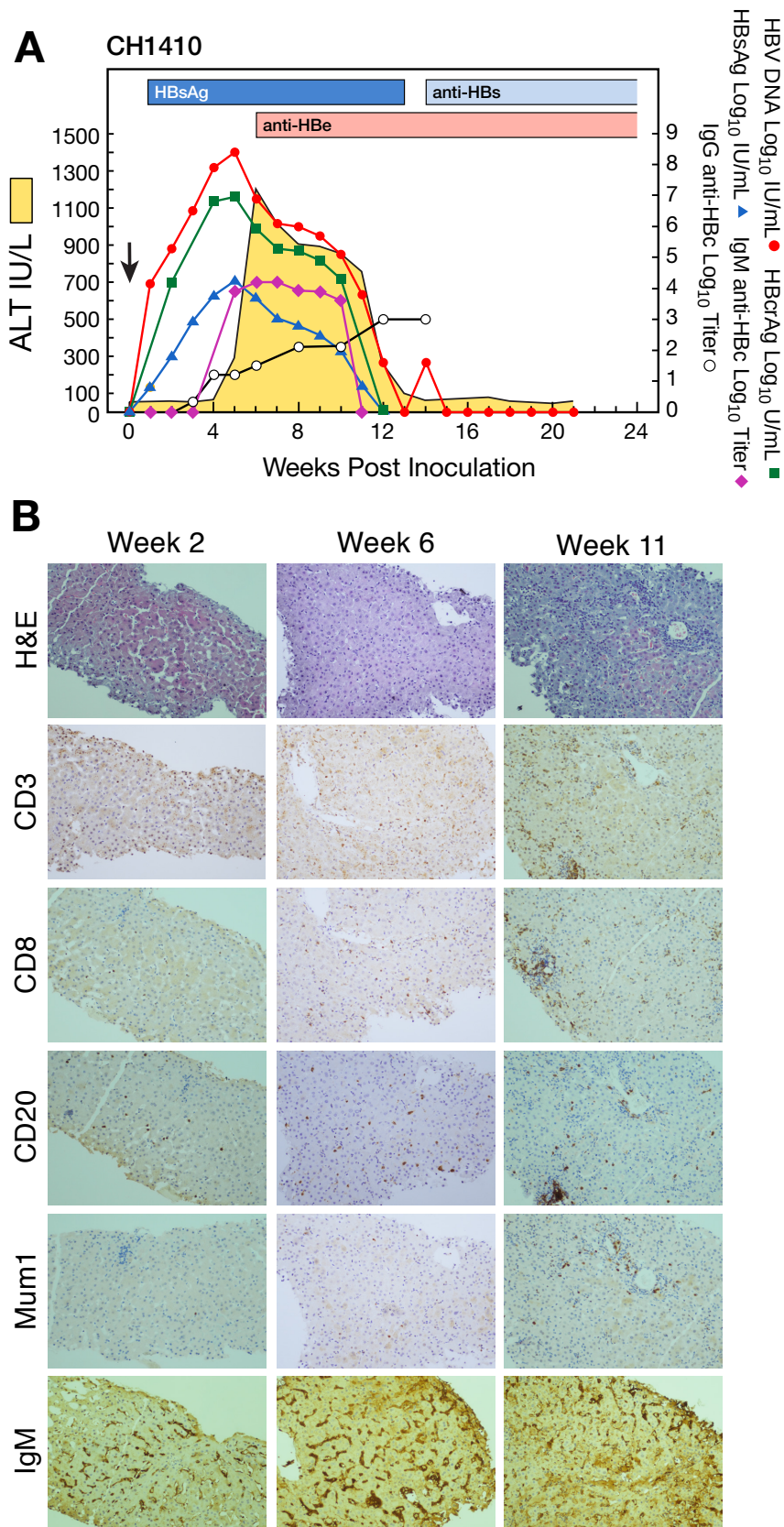

Supplement: S1 Fig — (A) Clinical, serologic and virologic course of severe AHB. (B) Hematoxylin and eosin (H&E) demonstrated extensive hepatocellular damage with hydropic swelling and acidophilic bodies when the ALT peaked at week 6, followed by the highest degree of necroinflammation after the ALT peak, at week 11. Immunohistochemical staining of T-cell and B-cell lineages in liver tissue at different time points during the course of HBV infection. Liver sections were stained with antibodies against CD3, CD8, CD20, Mum1/IRF4, and IgM. Images of liver sections (week 2 CD3 40X, all others 20X) show an infiltration by B and T cells initially distributed as single cells within the lobule at the time of the ALT peak, followed by an increase after the ALT peak with cells appearing both as single cells as well as clusters within the portal areas. There were also plasma cells positive for Mum1/IRF4 especially after the ALT peak along with rare plasma cells positive for IgM. Staining for IgM was predominantly confined to the sinusoids. (PDF) [file ppat.1008793.s001.pdf]
