# Supplementary material for: Distinct disease features in chimpanzees infected with a precore HBV mutant associated with acute liver failure in humans
Source: PLoS Pathog. 2020 Aug 31;16(8):e1008793. doi: 10.1371/journal.ppat.1008793 (PMC7485984; doi:10.1371/journal.ppat.1008793)
Supplement: S2 Fig — Numbers shown within circles are the total number of VH genes analyzed. Numbers shown in the overlapping area between circles represent shared sequences between chimpanzees. (PDF) [file ppat.1008793.s002.pdf]

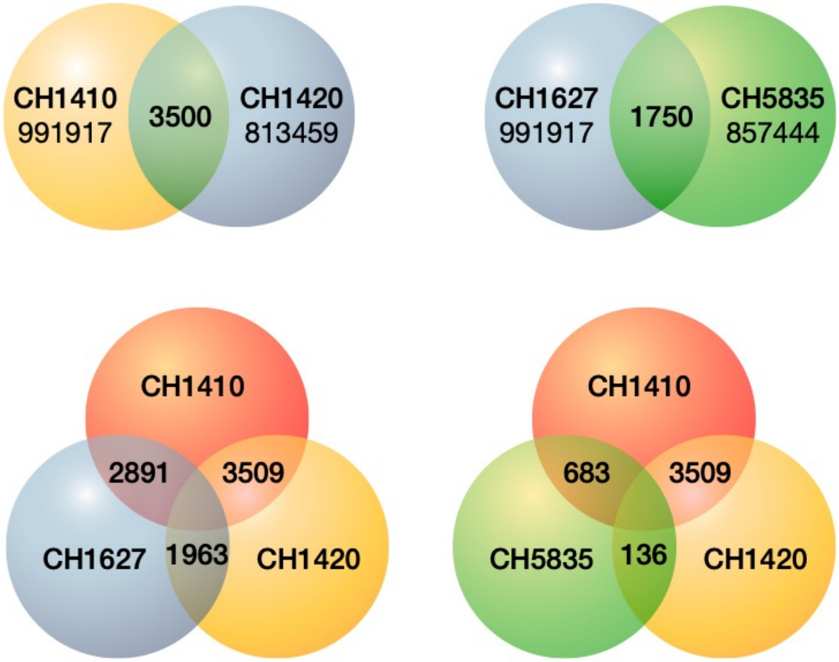

S2 Fig. Shared VH gene sequences amongst the four chimpanzees. Numbers shown within circles are the total number of VH genes analyzed. Numbers shown in the overlapping area between circles are numbers of shared sequences between chimpanzees.
