# Supplementary material for: Distinct disease features in chimpanzees infected with a precore HBV mutant associated with acute liver failure in humans
Source: PLoS Pathog. 2020 Aug 31;16(8):e1008793. doi: 10.1371/journal.ppat.1008793 (PMC7485984; doi:10.1371/journal.ppat.1008793)
Supplement: S1 Table — All the anti-HBcAg antibody genes identified in the phage-display libraries were found in the corresponding liver antibody repertoire analyzed by next-generation sequencing, and in most cases were highly expanded. (PDF) [file ppat.1008793.s003.pdf]

S1 Table. Expansion of anti-core antibody clones (>100 antibodies)

Anti-core antibody		Liver antibody repertoire				
Source	Antibody Name	Source	IGHV	IGHJ	HCDR3 Sequence (aa)	Size of Clone (No. Ab)
CH1410 WK6	A5	CH1410 WK7	V174-RF-AACZ040697481	IGHJ6	CTRASLDIVATISNFHGLTNYYYYMDVW	4287
	B2		V171-RF-AACZ040697471	IGHJ6	CAREMNLLYYYYYMDVW	2100
	C12		V184-RF-AACZ040698931	IGHJ4	CARDLTMVGVVIIPLDYW	1417
	F12		V184-RF-AACZ040698931	IGHJ3	CARDLTMVGVLIRAFDIW	977
	G6		V75-RF-AACZ040013231	IGHJ4	CTRGFSSETIYYNIWTGRKPSKNLEDKNFDYW	2782
	H10		V184-RF-AACZ040698931	IGHJ4	CARDFFSLWLEYGGLGYFDYW	1498
CH1410 WK12	A1	CH1410 WK12	V179-RF-AACZ040697491	IGHJ5	CARGRYLDIATAHSENWFDPW	2071
	A3		V0-RF-AACZ040013201	IGHJ4	CAKDRHSFRLRGPFDYW	1573
	A5		V174-RF-AACZ040697481	IGHJ6	CTRYNVDIVASISRYSGYNNKDYCYYMDVW	877
	B1		V75-RF-AACZ040013231	IGHJ4	CTRGFSSETIYYNIWTGRKPSKNLEDKNFDYW	1305
	B6		V181-RF-AACZ040697491	IGHJ2	CATVESEGGSWYSWYFDLW	944
	B7		V176-RF-AACZ040697481	IGHJ6	CARAPKDVDIVGAILSYPSRFSRDSYYYYMDVW	3145
	B9		V184-RF-AACZ040698931	IGHJ4	CARDFGFTWYYLDYW	270
	B10		V184-RF-AACZ040698931	IGHJ5	CARDSPLDIGGWPKACFDPW	1505
	C12		V179-RF-AACZ040697491	IGHJ4	CARGETVRLIYW	4631
	D6		V184-RF-AACZ040698931	IGHJ6	CVRGTCDLLHCPYYYMDVW	1815
	E3		V75-RF-AACZ040013231	IGHJ4	CTRGDYMVGGLIIYSLNFDNW	1653
	E7		V75-RF-AACZ040013231	IGHJ4	CTRGFSSDYGDSSYYLKLFDYW	761
	F6		V184-RF-AACZ040698931	IGHJ4	CARDFGFTYYYFDYW	236
	F10		V179-RF-AACZ040697491	IGHJ4	CARGRNSLDMVGVSQFEYW	2347
	G5		V179-RF-AACZ040697491	IGHJ2	CARDLSPELPVSQYLDWLTLGVYLW	128490
	G6		V184-RF-AACZ040698931	IGHJ6	CARDLQMVEIVVVPMDVW	1870
	G8		V179-RF-AACZ040697491	IGHJ2	CARDLSPELPVSQYLDWLTLGVYPW	744
	H10		V180-RF-AACZ040697491	IGHJ6	CTRDQELLSYYYYMDVW	4291
CH1420 WK10	B5	CH1420 WK11	V179-RF-AACZ040697491	IGHJ6	CARGRGVISYYMDVW	820
	D11		V85-RF-AACZ040013231	IGHJ6	CARVTLDIVATISAAPGYYYYYMDVW	114
	F9		V180-RF-AACZ040697491	IGHJ4	CTRARGRYCSSTSCYPYYFDYW	748
CH1627 WK14	D10-1		V170-RF-AACZ040697451	IGHJ3	CARGPTVAGTRSGAFDIW	2140
	F4		V83-RF-AACZ040013231	IGHJ6	CAREKRSTYYNYYYMDVW	501
CH1627 WK16	B3	CH1627 WK16	V174-RF-AACZ040697481	IGHJ4	CTRGPRRVDVVATIPRGYFDYW	1612
	B5		V75-RF-AACZ040013231	IGHJ4	CARGPKYSGYDPGFDYW	1398
	B12		V170-RF-AACZ040697451	IGHJ3	CARRHREHRAFDIW	776
	C4		V174-RF-AACZ040697481	IGHJ4	CTRGPRRVDIVATIPRGYFDYW	3062
	C6		V181-RF-AACZ040697491	IGHJ4	CATQRYSGYNFFFDYW	23437
	C10		V181-RF-AACZ040697491	IGHJ4	CATRGGTDYYEFYFDYW	4864
	C12		V75-RF-AACZ040013231	IGHJ4	CTRVSQYSGYDWARDW	8813
	D6		V174-RF-AACZ040697481	IGHJ4	CTRGPRRVDIVATIPRGYFDYW	3062
	D9		V181-RF-AACZ040697491	IGHJ4	CATRGGTDYYEFYFDYW	4864
	D10		V75-RF-AACZ040013231	IGHJ4	CSRGLSLWTGYYPGFDYW	602
	E5		V174-RF-AACZ040697481	IGHJ4	CTRGPRRVDVVATIPRGYFDYW	1612
	E11		V174-RF-AACZ040697481	IGHJ2	CTRERGVFCSGGSCYSPIIPGYFDLW	5366
	F3		V83-RF-AACZ040013231	IGHJ5	CARGLFGRKGYSNSWFDPW	1070
	G5		V170-RF-AACZ040697451	IGHJ3	CARGPTVAGTRSGAFDIW	2140
	G11		V75-RF-AACZ040013231	IGHJ4	CSRGEKRGNSGYSFPYYAYW	952
	H3		V83-RF-AACZ040013231	IGHJ5	CARGLFGRRGWTNSWFDPW	2351
	H7		V75-RF-AACZ040013231	IGHJ4	CTRVGPFPIVGTTLYFDYW	1446
CH5835 WK18	A1	CH5835 WK18	V75-RF-AACZ040013231	IGHJ4	CTRGRATVTTDQNAPNFGFW	2873
	A4		V170-RF-AACZ040697451	IGHJ4	CATEGGGRYTNFDYW	4639
	B6		V75-RF-AACZ040013231	IGHJ4	CARGPKYSGYDPGFDYW	829
	B8		V75-RF-AACZ040013231	IGHJ4	CSSLFRYFDYW	1374
	B9		V75-RF-AACZ040013231	IGHJ4	CTTGRYSGYDSQVLRDRYFDSW	305
	C4		V181-RF-AACZ040697491	IGHJ4	CATGHKYRGYDPGFDNW	991
	C5		V181-RF-AACZ040697491	IGHJ6	CATKASGDYGYYDHYMDVW	318
	C7		V170-RF-AACZ040697451	IGHJ5	CARWTSKGGYDLW	2327
	D7		V75-RF-AACZ040013231	IGHJ4	CTRTVRTVASWYDYW	271
	D9		V181-RF-AACZ040697491	IGHJ6	CTTQTSTVRTYYYYFMDVW	1677
	D10		V75-RF-AACZ040013231	IGHJ4	CTRGRSTTGTTPDSNFDYW	1173
	D12		V75-RF-AACZ040013231	IGHJ4	CTSGRDCTGGTCYAKLDSW	4338
	F4		V75-RF-AACZ040013231	IGHJ4	CSRGRSTTGTTPDSNFDYW	19429
	F5		V75-RF-AACZ040013231	IGHJ4	CTTGRDCSSGICYAKLDYW	3132
	G2		V181-RF-AACZ040697491	IGHJ4	CATLSRPGGYEFYFDYW	1561
	H4		V83-RF-AACZ040013231	IGHJ4	CARGRYDYITGTYRFGGAGTYYFDYW	7661
	H6		V75-RF-AACZ040013231	IGHJ4	CTRGRSLTGTTPDSSFDYW	13158
	H11		V75-RF-AACZ040013231	IGHJ4	CTRGRTTVATDQNGPNFDYW	193
